# Invertebrate community composition in dominant agroecosystems of Saskatchewan, Manitoba, and North Dakota

**DOI:** 10.1093/aesa/saaf037

**Published:** 2025-10-29

**Authors:** Mike M Bredeson, Alexandrea Michels, James O Eckberg, Steven T Rosenzweig, Kelton D Welch, Jonathan G Lundgren

**Affiliations:** Ecdysis Foundation, Blue Dasher Farm, Estelline, SD, USA; Ecdysis Foundation, Blue Dasher Farm, Estelline, SD, USA; General Mills, Food Systems, Golden Valley, MN, USA; General Mills, Food Systems, Golden Valley, MN, USA; Ecdysis Foundation, Blue Dasher Farm, Estelline, SD, USA; Ecdysis Foundation, Blue Dasher Farm, Estelline, SD, USA

**Keywords:** biodiversity, bioinventory, cropland, functional guild, intercrop

## Abstract

Agriculture currently occupies the majority of the former prairie ecosystem of North America, and farm management decisions affect the abundance and diversity of life in this region. Over 3 yr, we collected and identified the aboveground and soil insect communities from 159 agricultural fields representing 16 different food systems. A total of 107,320 specimens representing 990 operational taxonomic units were collected and identified. The invertebrate community was represented by 24 Orders, 189 Families, and 477 identified Genera. Herbivores were the most abundant (26,112 specimens; 59% of the non-Collembola/mite specimens) and taxonomically rich (308 species; 31%) functional group. Parasitoids, predators, omnivores, and detritivores were each represented by 8% to 10% of specimens collected. Perennial grassland habitats had the most abundant and diverse invertebrate communities (528 insects from 79 species per field); which was approximately twice that of any monocropped system. Including a second cash crop (ie intercropping) often doubled the insect community abundance and richness (380 specimens, 64 species) relative to the component monocrops. This research suggests that conservation and promotion of invertebrate biodiversity within agricultural landscapes may be fostered by supporting perennial grazing lands and intercropping as an alternative to monocrop farming.

## Introduction

Recently documented invertebrate declines have become emblematic of biodiversity loss on a planetary scale. Estimates of 30% to 45% declines in insect populations in the past 50 yr are supported by multiple scientific studies (Hallmann et al. 2017, Sanchez-Bayo and Wyckhuys 2019, Wagner et al. 2021, Isbell et al. 2023). These invertebrate declines are echoed in other terrestrial biological groups, including amphibians, birds, mammals, and plants (Sala et al. 2000, Moller 2019, Hendershot et al. 2020, Isbell et al. 2023). The rapid loss of biodiversity that Earth is experiencing increases the urgency that we inventory the life on earth as an important step in preserving and promoting what remains (Cardoso et al. 2011, Girardello et al. 2019). Invertebrate communities are an important indicator of habitat quality (Gerlach et al. 2013), but enumerating invertebrate diversity can be hampered by a lack of taxonomic expertise that can identify the species present (Cardoso et al. 2011). Nevertheless, bioinventories are an important tool for monitoring the relative impacts of land management decisions on biodiversity (Weisser et al. 2023).

Unintended consequences of land use change driven by agricultural production are major drivers of biodiversity loss (Sanchez-Bayo and Wyckhuys 2019, Zabel et al. 2019, Jaureguiberry et al. 2022). That said, degraded agroecosystems offer an opportunity for restoring biological diversity and ecosystem function in heavily agrarian regions. Agroecosystems currently occupy nearly 40% of the terrestrial surface of the planet (Raven and Wagner 2021). In North America’s Northern Great Plains, agroecosystems account for nearly 97% of land use, 80% of which is cultivated, and the remaining being primarily grazing lands (Shorthouse 2010, Willms et al. 2011). Conventionally cultivated ground is planted to a minimal number of crop species of narrow genetic diversity whose productivity is maintained with synthetic fertilizers and chemical pesticides (Carvalho 2017). Agricultural habitats with high disturbance further threaten biodiversity by opening ecological niches that invasive species exploit (Willms et al. 2011). While simplification of the agrobiome is associated with widespread species loss, the prevalence of agroecosystems makes conservation efforts in these habitats particularly important for increasing biodiversity and ecosystem function (Lockwood 1999, Kremen and Merenlender 2018, Hendershot et al. 2020, Kirk et al. 2025).

In response to growing recognition of agriculture’s pernicious environmental effects, land managers have begun to explore alternative strategies for producing food that align with rooted ecological principles. As they are termed, “Regenerative practices” show promise in increasing biodiversity in croplands while maintaining or improving the profitability and productivity of these habitats (LaCanne and Lundgren 2018, Fenster et al. 2021a,b, Lundgren et al. 2025). Too few comprehensive inventories of invertebrates in agroecosystems have been conducted (but see Lichtenberg et al. 2017). Limited taxonomic expertise, a tendency for insect researchers to collect in areas that are desirable to visit (Girardello et al. 2019), or perhaps for preferences to inventory taxonomic groups other than insects (Troudet et al. 2017) may support why more effort in sampling invertebrates of agroecosystems would be helpful.

The northern prairie pothole region associated with Manitoba and Saskatchewan in Canada, and northern North Dakota in the United States represents a formerly diverse mixed-grass prairie that has largely been converted to cropland (Wright and Wimberly 2013). Agricultural invertebrate communities change substantially over time (Fiser et al. 2025), making it helpful to have consistent and comprehensive inventories of these ecosystems. Our goals were to (i) describe the invertebrate species (especially insects) encountered in the aboveground and soil strata of major food systems of this region (species-level data are provided in Supplementary Table S1), and (ii) digitize the invertebrate collection for future research. Furthermore, we tested the hypothesis that different agroecosystems would support quantitatively distinct invertebrate communities.

## Materials and Methods

**Fig. 1.**
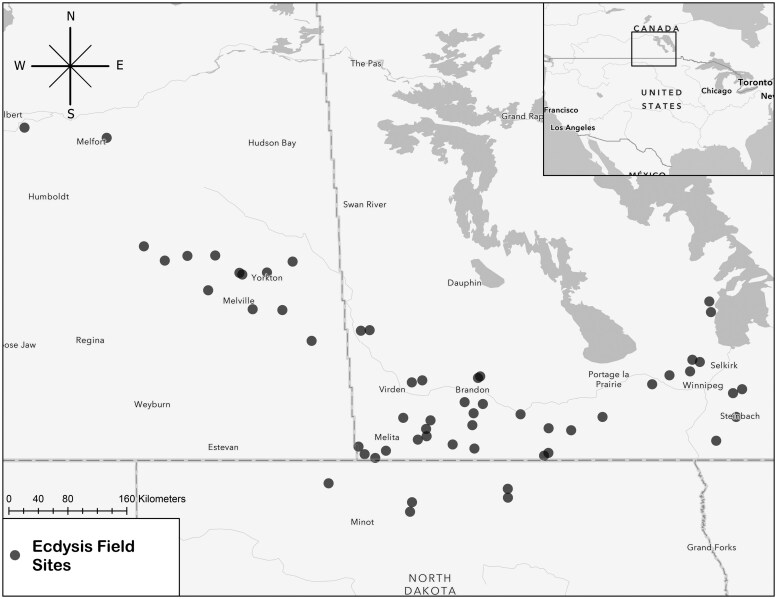
A map of the distribution of farms sampled in Saskatchewan and Manitoba, Canada and North Dakota, United States from 2019 to 2021.

Farms (average field size 65 ± 6 ha) representing a wide array of cropping systems and regenerative agriculture practice adoption were sampled in Saskatchewan, Manitoba, and North Dakota in 2019, 2020, and 2021. Participatory farmers volunteered their operations for study. Some of these fields were resampled annually throughout the study, but some were added (*n* = 8) or omitted *n* = 2) depending on farmer interest and resource availability. A total of 49, 55, and 55 farms were sampled in 2019, 2020, 2021, respectively (Fig. 1). Resampled fields grew entirely different crops and used different production methods in sequential years over the study period; this temporal aspect of the study was controlled for in the analysis, but was not a focus of the comparison of agroecosystems. Monocropped fields were indicative of dominant crops in the region, and included oats, spring wheat, canola, rye, peas, barley, soybeans, corn, and sunflowers (Table 1). Crops with fewer than 4 field observations were millet, flax, and black beans. Intercropped fields were those defined as having at least 2 cash crops that were produced simultaneously; intercrops observed in this study included 14 unique combinations (oat+pea, oat+sweet clover, oat+vetch, oat+barley, oat+rye, oat+pea+millet, canola+pea, wheat+faba, wheat+vetch, wheat+pea, wheat+red clover, flax+lentils, soy+barley, and triticale+peas). Cover cropped fields were those with a full season, annual cover crop that was planted in the spring of a sampling season, or in the preceding fall; occasionally these cover crops were grazed by cattle. Hay fields/silage were often perennial crops that were harvested for biomass (animal feed) that was moved off site. Perennial grasslands included planted pasture and native rangeland that often had high plant diversity that was allowed to persist for multiple years. Typically, these grasslands were grazed by cattle at least once per season. We targeted our sampling in the first half of July, when crop/pasture phenology corresponded with maximum biomass (eg crops attained full height), and crop height and density varied substantially among the different fields.

**Table 1.** Relative arthropod community descriptions in major food systems of Manitoba, Saskatchewan, and northern North Dakota

Food system	Total OTUs	*H*	*D*
**Barley (8)**	189	2.62±0.28 bcd	0.77±0.03
**Canola (13)**	224	2.79±0.16 abcd	0.78±0.03
**Corn (6)**	157	2.64±0.22 abcd	0.76±0.05
**Cover crop (9)**	229	2.78±0.29 abcd	0.78±0.03
**Hay/silage (13)**	306	2.93±0.18 ab	0.78±0.02
**Intercropped (26)**	527	3.06±0.09 a	0.75±0.01
**Oats (24)**	348	2.58±0.12 bcd	0.74±0.02
**Peas (8)**	176	2.40±0.23 cd	0.70±0.06
**Perennial grasslands (11)**	383	3.16±0.20 a	0.74±0.04
**Rye (10)**	167	2.35±0.14 d	0.73±0.05
**Soybean (5)**	129	2.89±0.18 abcd	0.88±0.03
**Sunflower (4)**	140	2.36±0.26 abcd	0.62±0.07
**Wheat (14)**	344	2.80±0.17 abc	0.75±0.02

The communities are combined across soil cores (3 cores) and sweep (150 sweeps) samples in each field. Numbers in parentheses following the food system descriptor represent the number of fields. In the species richness column, the numbers represent the total number of operational taxonomic units (OTUs) collected in that food system. The table presents 2 diversity indices: Shannon *H* and Simpson’s *D*. Values under the community parameters represent mean ± SEMs; values within a column followed by different letters are significantly different (Fisher’s LSD test; α = 0.07).

### Aboveground Arthropod Sampling

Aboveground arthropods were sampled via sweep net (50 sweeps per sample point; 38 cm net diameter, 91 cm handle length; SKU: 7625HS; Bioquip Products Inc, Rancho Dominguez, CA 90220, United States) at 3 sampling points within each field. Sampling points were marked with flagging ribbon along a transect at 50, 100, and 150 m from the field’s edge and in the direction toward a field’s interior. Care was taken to ensure that points along the transect were well representative of the field at-large and did not cross or come close to any alternative habitats such as waterways, tree belts, road ditches, wetlands, etc., which may host a unique arthropod community. Collections from each of the 3 distances along a transect were individually placed in labeled 1-gallon Ziploc bags (Item number- 314445; SC Johnson, Racine, WI 63403, United States). Invertebrates were euthanized and preserved by placing 5 ml of 70% isopropyl alcohol in sample bags. Samples were stored on ice in the field and then frozen at the laboratory until identification to the lowest taxonomic level possible.

### Subterranean Insect Sampling

Soil cores were used as a way to quickly collect densities of resident soil-dwelling insects in a single visit. Insects below the soil surface were collected at 50, 100, and 150 m along the established transect described above. At each distance, a single soil core (10 cm diameter × 10 cm deep) was extracted from an undisturbed area within the crop row using a golf hole cup cutter (SKU: RP1001; R&R Products, Tucson, AZ 85714, United States). Soil cores were placed in labeled 1-gallon Ziploc bags and stored on ice until they were returned to the laboratory.

### Extraction of Arthropods from Soil

In 2019, invertebrates were extracted from fresh (never frozen) soil cores via a Berlese funnel system beginning 60 h after field collection. Cores remained in the Berlese funnel system for 7 d. All collected invertebrates were stored in 70% isopropyl alcohol until identification. In study years 2020 and 2021, soil cores were collected in the same manner as in 2019, but soils containing invertebrates were frozen within 24 h after field collection. A floatation method based on previous work (Ladell 1936, Kethley 1991) was used to extract invertebrates. In this technique, a soil core was thawed, placed in a large circular metal pan (24 cm diameter, 10 cm deep), where large soil aggregates were broken apart by hand. Pilot studies revealed a difference in the abundances of some taxa recovered from floated samples versus those recovered from Berlese funnels, but similar overall diversities and abundances found in the communities from 2 extraction methods.

Magnesium sulfate (MgSO_4_) was used to separate the insects from the soil and detritus. Specifically, MgSO_4_ was dissolved in water to a specific gravity of 1.11, as measured using a manual hydrometer. This solution was used throughout the invertebrate extraction process and additional solution was mixed as needed. MgSO_4_ solution was poured into the pan containing a soil sample. This mixture was gently agitated until invertebrates and particulate organic matter floated to the surface and sand, silt, and clay fell to the container’s bottom. Additional MgSO_4_ solution was added to the pan until floating material nearly reached the pan’s upper rim.

A wire mesh screen (opening dimensions: 0.64 × 0.64 cm) was used to submerge pieces of organic matter down into the MgSO_4_ solution but allow small organic matter segments and invertebrates to float to the surface. Only the buoyant material was poured over a standard mesh window screen with a 53-micron sieve underneath (US Standard Sieve Series, #270, 53-microns; American Scientific Products, McGraw Park, IL 60085, United States). After an additional wash with clean MgSO_4_ solution, the material caught in the window screen was stored in a 50-ml vial with 70% ethanol. This large fraction was examined for large invertebrates under a dissecting microscope. Contents captured in the sieve were rinsed with water into a funnel (15 cm diameter, 10 cm high cylinder dimensions above a 45° funnel taper) equipped with a custom bottom control release valve. This fraction of small particulates and invertebrates was resuspended in 250 ml water and 25 ml soybean oil, then stirred vigorously. Once the invertebrate-containing oil layer had separated to the surface the lower water and debris was discharged. The remaining oil and invertebrates were released into a 53-micron sieve where oil was washed away with a spray of 99% isopropyl alcohol. Invertebrates were transferred to a 50-ml vial and stored with 70% isopropyl alcohol until examination under a dissecting microscope.

### Data Analysis

All specimens were identified microscopically to as low an operational taxonomic unit (OTU) as possible by first keying out specimens to family (Triplehorn et al. 2005), and then applying family-level keys and other resources to further resolve the identity of the specimens (Supplementary Doc1 contains a bibliography of resources used for identifying the taxonomy and functional groupings; https://doi.org/10.17605/osf.io/znhv4), and each was assigned a voucher number, and physical specimens are stored and publicly accessible at the Mark F. Longfellow Biological Collection at Blue Dasher Farm, Estelline, South Dakota 57234, United States. Additionally, images were taken at 12-Megapixel resolution and 16× to 80× magnification with a Flexacam C1 microscope camera mounted on a Leica M-125C microscope with Z-stacking capability. Vouchers are digitized on internally developed BugBox software (Welch and Lundgren 2024), which are complete and are publicly accessible (Bugbox 2025). Initially, all OTUs were assigned a voucher number, consisting of the Family and a 3-digit number (eg Figitidae 001). These voucher designators were replaced with genus and species epithets as available. When a genus had more than 1 OTU that could not be identified to species, they were designated as sp. 1, 2, etc. (eg *Ceraphron* sp. 1; *Ceraphron* sp. 2). Trophic function of invertebrates (based on primary trophic assignment) was assigned using the reference list (Supplementary Doc1). For the agroecosystem analysis, black beans, millet, and flax had < 4 observations and were omitted from the means comparisons. We also omitted mites and Collembola from the systems comparisons, as we did not have the expertise to separate these groups into OTUs. Shannon’s *H* invertebrate diversity was calculated for each study location. Pielou’s evenness metric (*J*) was also calculated as an additional community descriptor. A Chao1 estimator was used to measure the predicted number of OTUs found per field. There were several sites that had no doublets, and the adapted Chao1 formula was


$$N+{\;S}^{2}/2\left(D+1\right)),$$

where *N* is the number of taxa collected per field; *S* is the number of singletons, and *D* is the number of doubletons. To account for the fact that some of the fields were resampled over the 3-yr period, a linear mixed model with site as a random factor and crop type as a fixed factor, was used to determine whether significant differences in arthropod communities were present in the cropping systems. A Fisher’s LSD means separation was applied to those contrasts that revealed significantly different means. For the ANOVAs, α = 0.05, and for Fisher’s LSD, α = 0.07. To justify grouping the intercropped fields into a single treatment, we compared the invertebrate abundance and taxonomic richness among the different species combinations using an Kruskal-Wallis nonparametric ANOVA (none of these intercropped fields was resampled over the 3-yr study). The invertebrate community we present is large even under the most concise considerations; we opted to present an overview of the community found using the 2 sampling techniques, and then aggregated the community in the different agroecosystems (rather than presenting a soil and aboveground analysis for each). All statistical analysis was performed on Systat 13 (Grafiti LLC, Palo Alto, CA 94301, United States)

## Results

Over the 3 yr, we collected a total of 107,320 total specimens and OTU-level identifications are presented in Supplementary Table S1 (https://doi.org/10.17605/osf.io/znhv4). Arthropod species richness was 990 total OTUs across all agroecosystem types (Table 1; Supplementary Table S1). Over half of the total invertebrate abundance was soil mites (46,028 specimens) and Collembola (17,081 specimens) that were not identified further and are not included in the remaining discussion regarding macroarthropods. An average of 283.58 ± 22.57 macroarthropods were collected per field per year (48.27 ± 2.20 OTUs per field per year). This community was represented by 24 Orders and 189 Families. A total of 477 of the OTUs were identified to genus level, and 95 were identified to species level. A rarefaction analysis (Chao 1 estimator) revealed that there were a predicted 15.83 ± 1.49 species per sample per field in the soil, and our collection accounted for 58.36 ± 1.51% of these OTUs. In the aboveground community, our rarefaction analysis revealed a predicted 55.50 ± 2.43 OTUs, and our sampling accounted for 61.37 ± 1.23% of these taxa.

There were 259 OTUs (2,901 specimens) captured in the soil community, and 860 OTUs (41,342 specimens) captured in the aboveground community. Some of these taxa (133 OTUs) were captured in both the aboveground and soil communities. In the soil, flies, aphids, thrips, and pseudocentipedes were numerically dominant (>2% of the specimens). Specifically, OTUs that represented more than 2% of the soil community were *Psychoda* spp. (Diptera: Psychodidae; Psychodidae 004; *n* = 822), *Forcipomyia* sp. (Diptera: Ceratopogonidae; Ceratopogonidae 005; *n* = 228), *Lycoriella* (Diptera: Sciaridae; Sciaridae 005; *n* = 119), undifferentiated Thysanoptera (Thysanoptera; *n* = 108), Scutigerellidae 001 (Symphyla: Scutigerellidae; *n* = 86), Aphididae 001 (Hemiptera: Aphididae; *n* = 85), and Cecidomyiidae 005 (Diptera: Cecidomyiidae; *n* = 85). In plant foliage, aphids, flies, and plant bugs dominated the community numerically. More than 2% of the aboveground community were represented by *Delia* sp. (Diptera: Anthomyiidae; Anthomyiidae 001; *n* = 4,286), Aphididae 002 (Hemiptera: Aphididae; *n* = 2,839), *Incertella* sp. (Diptera: Chloropidae; Chloropidae 003; *n* = 2,302), *Macrosteles* sp. (Hemiptera: Cicadellidae; Cicadellidae 002; *n* = 2,092), *Apallates neocoxendix* (Diptera: Chloropidae; Chloropidae 005; *n* = 1,218), *Camptoprosopella slossonae* (Diptera: Lauxaniidae; Lauxaniidae 001; *n* = 1,095), *Aphis* sp. (Hemiptera: Aphididae; Aphididae 003; *n* = 1,046) and *Trigonotylus caelestialium* (Hemiptera: Miridae; Miridae 002; *n* = 988).

### Functional Groups of Arthropods

Herbivores dominated the arthropod community, being the most taxonomically rich and abundant functional group captured *n* = 308 species; 26,112 specimens) (Table 2). Parasitoids (*n* = 254) and predators (*n* = 185) were the next most taxonomically rich functional groups in these agroecosystems. In terms of abundance, parasitoids (4,294), omnivores (3,567), predators (3,513), and detritivores (3,460) were all frequently collected in these systems.

**Table 2.** Functional groups of arthropods collected in different agroecosystems of Saskatchewan, Manitoba, and northern North Dakota

Feeding guild	Number of taxa (specimens)
**Blood**	23 (2,086)
**Carrion**	10 (62)
**Detritivore**	79 (3,460)
**Dung**	21 (283)
**Herbivore**	308 (26,112)
**Omnivore**	42 (3,567)
**Parasitoid**	254 (4,294)
**Pollinator**	51 (511)
**Predator**	185 (3,513)
	973; 43,888

Specimens were either collected with soil cores or sweep nets.

### Communities in Different Agroecosystems

**Fig. 2.**
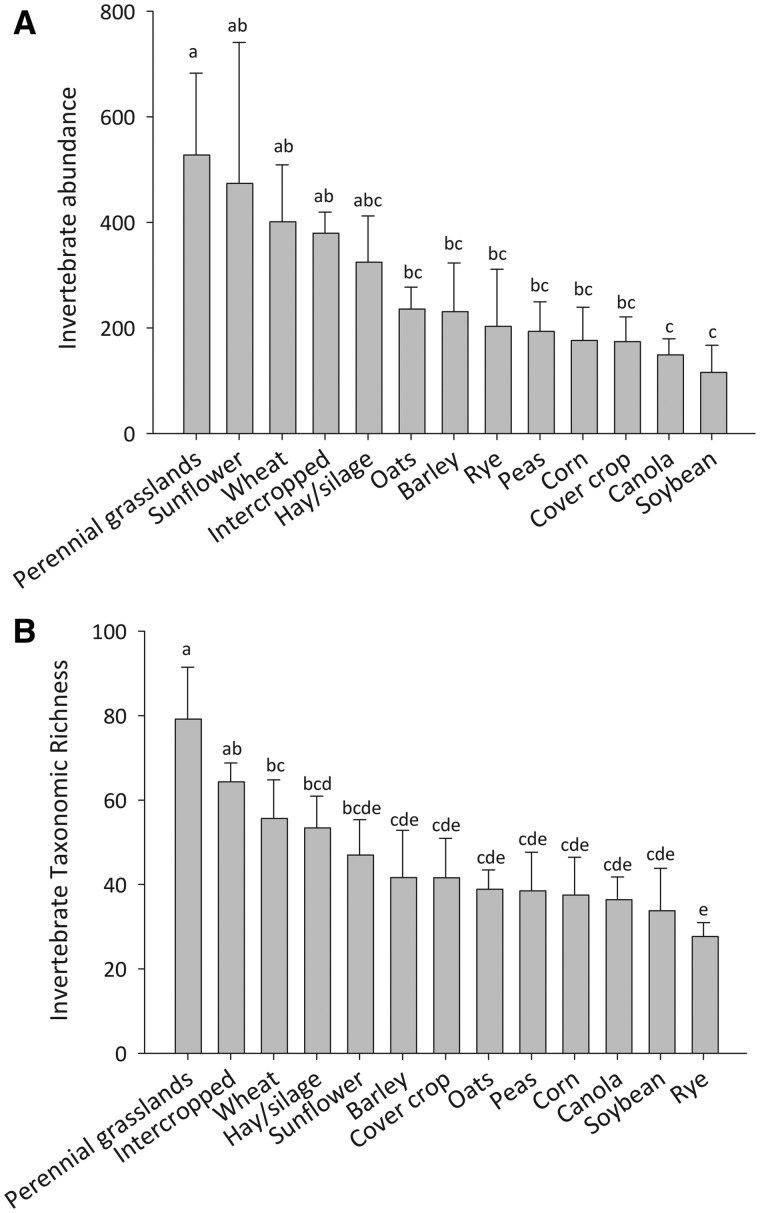
Relative arthropod abundance (A) and taxonomic richness (B) in major food systems of Manitoba, Saskatchewan, and northern North Dakota. The communities are combined across soil cores (3 cores) and sweep (150 sweeps) samples in each field. Bars represent means ± SEMs; values within a panel topped by different letters are significantly different (Fisher’s LSD test; α = 0.07).

Arthropod abundance (*F*_16, 86_ = 1.97, *P *= 0.04), taxonomic richness (*F*_16, 86_ = 2.42, *P *= 0.005), and taxonomic diversity (*F*_16, 84_ = 2.00, *P *= 0.02) differed among agroecosystems (Table 1; Fig. 2). Pielou’s evenness metric (*J*) was statistically similar among the agroecosystems (*F*_16, 81_ = 099; *P *= 0.48). The Fisher’s LSD post hoc comparisons showed that arthropod abundance was greater in perennial habitats (perennial grassland systems, hay/silage systems) and these habitats also possessed the most diverse and abundant invertebrate communities. Intercropped fields with different crop species combinations had similar soil invertebrate abundance (χ^2^_14_ = 13.82, *P *= 0.46), richness (χ^2^_15_ = 14.99, *P *= 0.45), and Shannon *H* (χ^2^_15_ = 13.38, *P *= 0.57). Likewise, intercropped systems with different crop species had similar aboveground invertebrate abundance (χ^2^_15_ = 18.29, *P *= 0.24), richness (χ^2^_15_ = 16.74, *P *= 0.36), and Shannon *H* (χ^2^_15_ = 15.09, *P *= 0.45). Intercropped systems showed more richness than most cropping systems and greater abundance than some cropping systems. Wheat and sunflowers generally showed greater abundance and diversity than the cropping systems with the most sparse insect communities.

## Discussion

This study revealed that agroecosystems of the northern prairie of North America can harbor an abundant and rich invertebrate community. With nearly 1,000 taxonomic records from 16 different habitats in the region, this community assessment is one of the more comprehensive descriptions of these communities conducted in recent years. Agroecosystems of this region harbored between 121-527 OTUs of invertebrates per field, which is an underestimate as we did not further refine some speciose groups like Collembola and Acari (mites). Elsewhere, regional and local assessments estimate that approximately 100 to 500 different species are found in individual row-cropping agroecosystems of the Plains Region of North America and in similar climates worldwide (Pimentel and Wheeler 1973, de la Fuente et al. 2003, Caballero-Lopez et al. 2010, Lundgren et al. 2013, Bredeson and Lundgren 2015, Lundgren et al. 2015, Welch and Lundgren 2016), and 350 to 3,000 species in native prairie and perennial habitats of this climatic zone (Nagel 1979, Tscharntke and Greiler 1995, Kaufman et al. 1998, Fay 2003, Schmid et al. 2015). These numbers are supported by the work presented here.

Applying additional sampling methods and expanding the sampling effort in space and time would have added to our perception of the community here. Employing additional sampling methods might reveal even more species from these habitats. Pitfall traps (including barrier-linked pitfall traps) are an excellent way to catch large numbers of taxa (Lundgren et al. 2009, Saska et al. 2013), particularly of larger taxa such as carabid beetles (Melnychuk et al. 2003). Malaise traps and optical sensors can catch large numbers of aerial insects that may be using a focal habitat (Rydhmer et al. 2024). And colored bee bowls are effective for capturing bees, flies, and other insect groups (Mogren et al. 2016). Also, it should be noted that this was a single snapshot of each of these fields per year. It is possible that some foliar herbivores (eg thrips and aphids) found in the soil samples may have occurred when the insects fell from plants during sampling. Additional sampling across the season, using a broader suite of sampling approaches would involve even greater biodiversity associated with these habitats. Also, this is a systems-level project that was conducted on operating farms that represented a diversity of different habitat characteristics. While crop was a consistent classification used to examine these invertebrate communities, we recognize that crop species-specific habitat characteristics may have been important mechanisms that influenced the observed patterns.

The distribution of invertebrate abundance within functional groups in these agroecosystems was consistent with patterns revealed in many invertebrate communities. The majority of insect specimens (59.4%) and the dominant fraction of insect OTUs (31.6%) found in these agroecosystems were herbivorous. Harper (1988) found 48% of species from these habitats to be herbivores (Seastedt 1984). Bredeson and Lundgren (2015) found that 52% of aboveground insects in sunflowers of South Dakota were herbivores. Even so, very few of these herbivores were economically threatening to a farm’s operation. Herbivores become “pests” when they reach a certain abundance threshold that justifies economic investment in their suppression. For example, 17 species of Miridae were collected in these agroecosystems, and only one of these species (*Lygus. hesperus*, representing < 25% of mirid specimens) is known to be a pest of crops under some conditions. Likewise, 23 species of Curculionidae were collected in these crop fields (248 individuals), and none of them are pest species on these crops. Worldwide, if approximately 1,000 to 10,000 insect species are considered economically threatening to humans (ie “pests”) (Triplehorn et al. 2005), and there are estimated to be 5.5 million insect species (Stork 2018), then for every pest species on earth there are between 550 and 5,500 species of insects that are beneficial, or at least not directly harmful, to humanity. Our estimates from Northern Plains agroecosystems fall within this predicted range. Our work found that after herbivores, parasites, predators, and detritivores were the next most abundant functional groups, which is similar to previous bioinventories (Harper 1988). If we were to include detritivorous Collembola in this analysis (Ke and Scheu 2008), the abundance of this guild would have inflated. The observed distribution of invertebrate functional groups have been appreciated for multiple decades (Southwood 1973) and follows a well-established patterns of lower trophic level animals exhibiting higher abundance and biomass relative to those in high trophic positions.

Perennial habitats harbored the most diverse and abundant invertebrate communities. Our observations were that perennial grassland fields had at least twice the abundance and species richness of most annual cropping system assessed (Table 2). Perennial habitats tend to have greater plant diversity and greater habitat complexity than annual agroecosystems, both necessary requirements for harboring a diverse and abundant community of higher trophic organisms (Tscharntke and Greiler 1995). These perennial grassland systems also coevolved with an associated invertebrate fauna, and this could have led to greater diversification in this community. Hay fields were another example of how perennial systems can support greater species richness than many simplified monocropping scenarios. Alfalfa has even been elevated in agri-environmental schemes as a way to promote biodiversity (Gonzalez del Portillo et al. 2022). Though more species-rich than many monocrops, we hypothesize that increased disturbance (eg cutting and removing plant biomass; Summers 1976, Hossain et al. 2002) of hay fields may have reduced taxonomic richness relative to perennial grazing lands.

Intercropping—simply adding a second crop species to an annual cropland scenario—also increased invertebrate communities. Intercropped fields had nearly twice as many OTUs and at least 1.5 times the abundance of invertebrates found in monocropped fields. The aggregation of different intercrop scenarios justified inclusion as a treatment in that we saw similar invertebrate communities in the different crop combinations, suggesting that these are not species-specific outcomes, but rather an outcome of doubling the number of crops in the agroecosystem. Exceptions were wheat and sunflowers, which both had robust insect communities with similar abundance and diversity to intercropped fields, something that has been seen in at least one other study of sunflowers as intercrops (de la Fuente et al. 2014). This observation is possibly a result of sunflower’s native status and winter wheat’s longer establishment period as a fall-planted, winter hardy plant. Other studies have also found that invertebrate populations are increased in intercropped fields (Slosser et al. 2000, Lundgren 2009, Alarcón-Segura et al. 2022, Huber et al. 2022, Pierre et al. 2022, Jarvinen et al. 2023). Changes in land management have selected for agrobiont species that thrive primarily in agricultural habitats (Samu and Szinetár 2002, Mogren et al. 2016). By increasing the number of crops in a field, it appears that this accumulated not only the habitat specialists for these 2 crops. We hypothesize that the high number of total OTUs found in the intercropped category of agroecosystems is a reflection of the multiple intercropping combinations, and associated habitat specialists in each, that are present in this agroecosystem grouping.

## Supplementary Material

saaf037_Supplementary_Data

## Data Availability

Data from this study are available from the Open Science Framework https://doi.org/10.17605/OSF.IO/ZNHV4
