# Supplementary material for: Invertebrate community composition in dominant agroecosystems of Saskatchewan, Manitoba, and North Dakota
Source: Ann Entomol Soc Am. 2025 Oct 29;119(1):38–45. doi: 10.1093/aesa/saaf037 (PMC12815888; doi:10.1093/aesa/saaf037)
Supplement: saaf037_Supplementary_Data [file saaf037_supplementary_data.zip › Supplementary-Doc1.docx]

Allen, R. T. (2002). A synopsis of the Diplura of North America: Keys to higher taxa, systematics, distributions and descriptions of new taxa (Arthropoda: Insecta). *Transactions of the American Entomological Society (1890-)*, *128*(4), 403–466.

Anderson, R. S., & Anderson, R. S. (1987). Systematics, phylogeny and biogeography of New World weevils traditionally of the tribe Cleonini (Coleoptera: Curculionidae: Cleoninae). *Quaestiones Entomologicae*, *23*(4), 431–710.

Artigas, J. N. (1966). The genus *Diogmites* (robber flies) in eastern United States (Diptera: Asilidae). *The Ohio Journal of Science*, *66*(4), 401–421.

Bailey, S. F., & Cott, H. E. (1954). A review of the genus *Heterothrips* Hood (Thysanoptera: Heterothripidae) in North America, with descriptions of two new species. *Annals of the Entomological Society of America*, *47*(4), 614–635.

Ball, E. D., & Ball, E. D. (1935). The genus *Bruchomorpha* Newman (Homoptera-Fulgoridae). *Bulletin of the Brooklyn Entomological Society*, *30*, 197–203.

Barber, H. G. (1935). New *Geocoris* from the United States, with key to species (Lygæidæ: Geocorinæ). *Journal of the New York Entomological Society*, *43*(2), 131–137.

Barnes, J. (2010). The Genus *Atomosia* MacQuart (Diptera: Asilidae) in North America north of Mexico. *Proceedings of the Entomological Society of Washington*, *110*, 701–732.

Bartlett, C. R., O’Brien, L. B., & Wilson, S. W. (2014). A review of the planthoppers (Hemiptera: Fulgoroidea) of the United States. Sheridan Books. https://www.biodiversitylibrary.org/page/51568500

Bergeron, M. D., Marshall, S. A., & Swann, J. E. (2015). A review of the New World *Coproica* (Diptera: Sphaeroceridae) with a description of 8 new species. *Zootaxa*, *3953*(1), Article 1.

Bishop, S. C., & Crosby, C. R. (1932). Studies in American spiders: The genus *Grammonota*. *Journal of the New York Entomological Society*, *40*(4), 393–421.

Bohart, R. M., & Menke, A. S. (1963). A reclassification of the Sphecinae: With a revision of the Nearctic species of the tribes Sceliphronini and Sphecini (Hymenoptera, Sphecidae). *University of California Publications in Entomology*, *30*(2), 91–182.

Borror, D. J. (1945). A key to the New World genera of Libellulidae (Odonata). *Annals of the Entomological Society of America*, *38*(2), 168–194. https://doi.org/10.1093/aesa/38.2.168

Bouchard, J. R. (2004). Guide to aquatic macroinvertebrates of the upper Midwest waters. Water Resources Center, University of Minnesota.

Boucher, S. (2002). Revision of Nearctic species of *Cerodontha* (Cerodontha) (Diptera: Agromyzidae). *The Canadian Entomologist*, *134*, 1–27.

Boucher, S. (2003). The new world species of *Cerodontha* (Xenophytomyza) Frey (Diptera: Agromyzidae). *Zootaxa*, *178*(1), Article 1.

Boucher, S. (2012). Revision of the Nearctic species of *Cerodontha* (*Icteromyza*) (Diptera: Agromyzidae). *The Canadian Entomologist*, *144*(1), 122–157.

Bousquet, Y., & Laplante, S. (1999). Taxonomic review of the Canadian species of the genus *Monotoma* Herbst (Coleoptera: Monotomidae). *Proceedings of The Entomological Society of Ontario*, *130*, 67–96.

Brailovsky, H. (2015). A new genus and species of *Micropterous* Blissidae (Hemiptera: Heteroptera: Lygaeoidea) from Ecuador. *Zootaxa*, *4028*(4), Article 4.

Brailovsky, H. (2016). A review of the Geocoridae of Mexico (Hemiptera: Heteroptera: Lygaeoidea), with descriptions of four new species, new distributional records, and a key to the known subfamilies, tribes, genera and species. *Zootaxa*, *4173*(5), Article 5.

Bright, D. E. (1994). Revision of the genus *Sitona* (Coleoptera: Curculionidae) of North America. *Annals of the Entomological Society of America*, *87*(3), 277–306.

Brown, W. J. (1932). The North America species of *Glischrochilus* (Coleop.). *The Canadian Entomologist*, *64*(11), 255–262.

Buck, M., & Marshall, S. A. (2009). Revision of new world *Leptocera* Olivier (Diptera, Sphaeroceridae). *Zootaxa*, *2039*(1), Article 1.

Buck, M., Marshall, S. A., & Cheung, D. K. B. (2008). Identification atlas of the Vespidae (Hymenoptera, Aculeata) of the northeastern Nearctic region. *Canadian Journal of Arthropod Identification*, *5*, 1–492.

Burt, T. (2015). Taxonomic revision of four Nearctic Conopidae (Insecta: Diptera) genera with notes on all other Nearctic genera [Doctoral Dissertation, Carleton University]. https://repository.library.carleton.ca/concern/etds/jm214p89c

Campbell, J. M. (1975). A revision of the genera *Coprophorus* and *Cilea* (Coleoptera: Staphylindae) of America north of Mexico. *The Canadian Entomologist*, *107*(2), 175–216.

Carpenter, F. M. (1940). A revision of the Nearctic Hemerobiidae, Berothidae, Sisyridae, Polystoechotidae and Dilaridae (Neuroptera). *Proceedings of the American Academy of Arts and Sciences*, *74*(7), 193–280.

Carpenter, J. M. (2004). *Ancistroceroides* de Saussure, a potter wasp genus new for the United States, with a new key to the genera of Eumeninae of America north of Mexico (Hymenoptera: Vespidae). *Journal of the Kansas Entomological Society*, *77*(4), 721–741.

Cartwright, O. L. (1974). *Ataenius, Aphotaenius*, and *Pseudataenius* of the United States and Canada (Coleoptera: Scarabaeidae: Aphodiinae). Smithsonian Institution Press. http://repository.si.edu/xmlui/handle/10088/5385

Chamberlin, R. V. (1935). Miscellaneous new American spiders. *Bulletin of the University of Utah*, *26*(4), 1–79.

Chandler, D. S. (2005). A revision of the new world *Cyclodinus* Mulsant & Rey (Coleoptera: Anthicidae). *Transactions of the American Entomological Society (1890-)*, *131*(1/2), 1–20.

Chani-Posse, M. (2014). An illustrated key to the new world genera of *Philonthina* Kirby (Coleoptera: Staphylinidae), with morphological, taxonomical and distributional notes. *Zootaxa*, *3755*(1), Article 1.

Chen, H., Masner, L., & Johnson, N. F. (2017). New world species of the genus *Calliscelio* Ashmead (Hymenoptera, Platygastridae, Scelioninae). *ZooKeys*, *648*, 1–136.

Crosby, C. R., Seeley, R. M., & Bishop, S. C. (1928). Revision of the spider genera *Erigone*, *Eperigone* and *Catabrithorax* (Erigoneae). *New York State Museum Bulletin*, *278*, 1–98.

Cushing, P. E., & Roth, V. (2005). Spiders of North America: An identification manual (D. Ubick & P. Paquin, Eds.; 1st ed.). American Arachnological Society. http://catdir.loc.gov/catdir/enhancements/fy1208/2012392590-b.html

Darling, D. C., & Werren, J. H. (1990). Biosystematics of *Nasonia* (Hymenoptera: Pteromalidae): Two new species reared from birds’ nests in North America. *Annals of the Entomological Society of America*, *83*(3), 352–370.

Davis, J. C. (1970). Revision of the genus *Blapstinus* (Sturm) of America north of Mexico with notes on extralimital species (Coleoptera: Tenebrionidae) [Doctoral Dissertation, The Ohio State University]. https://www.proquest.com/openview/c06b52c14eb41072e8479f804977ca01/1?cbl=18750&diss=y&pq-origsite=gscholar

DeLong, D. M. (1949). The leafhoppers, or Cicadellidae, of Illinois (Eurymelinae, Balcluthinae). *Illinois Natural History Survey Bulletin*, *24*(1–4), Article 1–4.

Dietrich, C. H. (1994). Systematics of the leafhopper genus *Draeculacephala* Ball (Homoptera: Cicadellidae). *Transactions of the American Entomological Society (1890-)*, *120*(2), 87–112.

Dietrich, C. H. (2005). Keys to the families of Cicadomorpha and subfamilies and tribes of Cicadellidae (Hemiptera: Auchenorrhyncha). *Florida Entomologist*, *88*(4), 502–517.

Doering, K. C. (1932). The Genus *Acanalonia* in America north of Mexico (Fulgoridae, Homoptera). *Annals of the Entomological Society of America*, *25*(4), 758–786.

Dondale, C. D., & Redner, J. H. (1978). Revision of the Nearctic wolf spider genus *Schizocosa* (Araneida: Lycosidae). *The Canadian Entomologist*, *110*(2), 143–181.

Duperre, N. (2013). Taxonomic revision of the spider genera *Agyneta* and *Tennesseellum* (Araneae, Linyphiidae) of North America north of Mexico with a study of the embolic division within Micronetinae sensu Saaristo & Tanasevitch 1996. *Zootaxa*, *3674*, 1.

Eason, E. H. (1982). A review of the north-west European species of Lithobiomorpha with a revised key to their identification. *Zoological Journal of the Linnean Society*, *74*(1), 9–33.

Edmonds, W., & Zídek, J. (2012). Taxonomy of *Phanaeus* revisited: Revised keys to and comments on species of the new world dung beetle genus *Phanaeus* MacLeay, 1819 (Coleoptera: Scarabaeidae: Scarabaeinae: Phanaeini). *Insecta Mundi, 274*, 1-108. https://digitalcommons.unl.edu/insectamundi/784

Edwards, C. A. (1959). Keys to the genera of the Symphyla. *Zoological Journal of the Linnean Society*, *44*(296), 164–169.

Evans, H. E. (1961). A preliminary review of the Nearctic species of Sierolomorpha (Hymenoptera). *Breviora*, *140*, 1–12.

Eyles, A. C., & Schuh, R. T. (2003). Revision of New Zealand Bryocorinae and Phylinae (Insecta: Hemiptera: Miridae). *New Zealand Journal of Zoology*, *30*(3), 263–325.

Fall, H. C. (1912). A review of the North American species of *Collops* (Col.). *Journal of the New York Entomological Society*, *20*(4), 249–274.

Fedoseeva, L. I. (2004). Chloropid flies of the genus *Meromyza* Meigen, 1830 (Diptera: Chloropidae) of the fauna of North America. *Russian Entomological Journal*, *12*(4), 425–430.

Ford, E. J. (1996). The genus *Stelidota* Erichson in North America: A new species from Florida, new synonymy and lectotype designations (Coleoptera: Nitidulidae). *The Coleopterists Bulletin*, *50*(2), 149–153.

Forshage, M., & Nordlander, G. (2008). Identification key to European genera of Eucoilinae (Hymenoptera, Cynipoidea, Figitidae). *Insect Systematics & Evolution*, *39*(3), 341–359.

Foster, G. A., & Mathis, W. N. (2003). A revision of the genera Pelomyia Williston and Masoniella Vockeroth (Diptera:Tethinidae). Smithsonian Institution Press.

Fouts, R. M. (1948). Parasitic wasps of the genus *Trimorus* in North America. *Proceedings of the United States National Museum*, *98*(3225), 91–148.

Fracker, S. B. (1918). The Alydinae of the United States. *Annals of the Entomological Society of America*, *11*, 255–280.

Froeschner, R. C., & Froeschner, R. C. (1960). Cydnidae of the Western Hemisphere. *Proceedings of the United States National Museum*, *111*(3430), 337--680.

Galli, L., Shrubovych, J., Bu, Y., & Zinni, M. (2018). Genera of the Protura of the world: Diagnosis, distribution, and key. *ZooKeys*, *772*, 1–45.

Génier, F. (1989). A revision of the genus *Hoplandria* Kraatz of America North of Mexico (Coleoptera: Staphylinidae, Aleocharinae). *The Memoirs of the Entomological Society of Canada*, *121*(S150), 3–59.

Gibson, E. H. (1919). The genus *Gargaphia* Stål (Tingidae; Heteroptera). *Transactions of the American Entomological Society (1890-)*, *45*(3), 187–201.

Gibson, G. A., Huber, J. T., & Woolley, J. B. (Eds.). (1997). Annotated keys to the genera of Nearctic Chalcidoidea (Hymenoptera). NRC Research Press. 794 p.

Gittins, A. R. (1969). Revision of the Nearctic Psenini (Hymenoptera: Sphecidae) I. redescriptions and keys to the genera and subgenera. *Transactions of the American Entomological Society (1890-)*, *95*(1), 49–76.

Goodnight, C. J., & Goodnight, M. L. (1942). The genus *Protolophus* (Phalangida). *American Museum Novitates*, *1157*, 1–8.

Goulet, H. & Huber, John T. (Eds.). (1993). Hymenoptera of the world: An identification guide to families. Agriculture Canada. 668 p.

Green, J. W. (1966). Revision of the Nearctic species of *Silis* (Cantharidae: Coleoptera). *Proceedings of the California Academy of Sciences*, *32*, 447–513.

Grzywacz, A., Hall, M. J. R., Pape, T., & Szpila, K. (2017). Muscidae (Diptera) of forensic importance—An identification key to third instar larvae of the western Palaearctic region and a catalogue of the muscid carrion community. *International Journal of Legal Medicine*, *131*(3), 855–866.

Gusarov, V. I. (2003). Revision of some types of North American Aleocharines (Coleoptera: Staphylinidae: Aleocharinae), with synonymic notes. *Zootaxa*, *353*(1), 1.

Guzman-Larralde, A., Leyva, J., & Valdez, J. M. (2001). Illustrated key to genera of Mymaridae (Hymenoptera) of central Mexico. *Southwestern Entomologist*, *26*, 245–252.

Hackman, Walter. (1959). On the genus *Scaptomyza* Hardy (Dipt., Drosophilidae). *Acta Zoologica Fennica*, *97*, 1–73.

Hamilton, K. G. A. (1979). Synopsis of the North American Philaenini (Rhynchota: Homoptera: Cercopidae) with a new genus and four new species. *The Canadian Entomologist*, *111*(2), 127–141.

Hamilton, K. G. A. (2002). The spittlebugs of Canada: Homoptera: Cercopidae. Agriculture Canada. 108 p. https://publications.gc.ca/site/eng/9.811354/publication.html

Hardy, D. E. (1944). Revision of Nearctic Bibionidae including Neotropical *Plecia* and *Penthetria* (Diptera). *University of Kansas Science Bulletin*, *30*(15), 367–555.

Hardy, D. E. (1949). The North American *Chrysopilus* (Rhagionidae-Diptera). *The American Midland Naturalist*, *41*(1), 143–167.

Hayat, M. (1983). The genera of Aphelinidae (Hymenoptera) of the world. *Systematic Entomology*, *8*(1), 63–102.

Herbert, D. A. J., Kamminga, K., Malone, S. M., Kuhar, T. P., Day, E. R., Greene, J. K., Brown, L., & Ellsworth, P. C. (2014). Field Guide to Stink Bugs of Agricultural Importance in the United States (2nd ed.). Virginia Cooperative Extension. 40 p. http://hdl.handle.net/10919/75478

Herring, J. L. (1966). The genus *Orius* of the Western Hemisphere (Hemiptera: Anthocoridae). *Annals of the Entomological Society of America*, *59*(6), 1093–1109.

Hoffman, R. L. (1996). Seed Bugs of Virginia. *The Insects of Virginia*, *14*, 1–118.

Hopper, K., Woolley, J., Hoelmer, K., Wu, K., Qiao, G.-X., & Lee, S. (2012). An identification key to species in the *mali* complex of *Aphelinus* (Hymenoptera, Chalcidoidea) with descriptions of three new species. *Journal of Hymenoptera Research*, *26*, 73–96.

Huber, J. T., Read, J. D., & Triapitsyn, S. V. (2020). Illustrated key to genera and catalogue of Mymaridae (Hymenoptera) in America north of Mexico. *Zootaxa*, *4773*(1), Article 1.

Ivie, W. (1969). North American spiders of the genus *Bathyphantes* (Araneae, Linyphiidae). *American Museum Novitates*, no. 2364.

James, M. T. (1939). A review of the Nearctic Beridinae (Diptera, Stratiomyidae). *Annals of the Entomological Society of America*, *32*(3), 543–548.

James, M. T. (1950). The genus *Scopeuma* in the western United States and southwestern Canada (Diptera, Scopeumatidae). *Annals of the Entomological Society of America*, *43*(3), 343–353.

Johnson, D. L. (2002). Spur-throated grasshoppers of the Canadian Prairies and Northern Great Plains. *Arthropods of Canadian Grasslands*, *8*, 16–25.

Kathirithamby, J., & Taylor, S. J. (2005). A new species of *Halictophagus* (Insecta: Strepsiptera: Halictophagidae) from Texas, and a checklist of Strepsiptera from the United States and Canada. *Zootaxa*, *1056*(1), Article 1.

Keirans, J. E., & Litwak, T. R. (1989). Pictorial key to the adults of hard ticks, family Ixodidae (Ixodida: Ixodoidea), east of the Mississippi River. *Journal of Medical Entomology*, *26*(5), 435–448.

Kelton, L. A. (1971). Revision of the species of *Trigonotylus* in North America (Heteroptera: Miridae). *The Canadian Entomologist*, *103*(5), 685–705.

Kelton, L. A. (1975). The lygus bugs (Genus *Lygus* Hahn) of North America (Heteroptera: Miridae). *The Memoirs of the Entomological Society of Canada*, *107*(S95), 5–101.

Kimsey, L. S. (2009). Taxonomic purgatory: Sorting out the wasp genus *Myzinum* Latreille in North America (Hymenoptera, Tiphiidae, Myzininae). *Zootaxa*, *2224*(1), 1.

Kimsey, L. S. (2014). California cuckoo wasps in the family Chrysididae (Hymenoptera). University of California Press. 318 p. https://escholarship.org/uc/item/3j6466jw

Kingsolver, J. M. (2004). Handbook of the Bruchidae of the United States and Canada (Insecta, Coleoptera) (Vol. 1). U.S. Department of Agriculture, Agricultural Research Service.340 p.

Kirk, K., & Bomar, C. R. (2005). Guide to the Grasshoppers of Wisconsin. Bureau of Integrated Science Services. 154 p.

Klimaszewski, J., Webster, R. P., Bourdon, C., Pelletier, G., Godin, B., & Langor, D. W. (2015). Review of Canadian species of the genus *Mocyta* Mulsant & Rey (Coleoptera, Staphylinidae, Aleocharinae), with the description of a new species and a new synonymy. *ZooKeys*, *487*, 111–139.

Klimaszewski, J., Webster, R., Savard, K., & Couture, J. (2009). First record of the genus *Alisalia* Casey from Canada, description of two new species, and a key to all Nearctic species of the genus (Coleoptera, Staphylinidae, Aleocharinae). *ZooKeys*, *25*, 1–18.

Klymko, J., & Marshall, S. A. (2008). Review of the Nearctic Lonchopteridae (Diptera), including descriptions of three new species. *The Canadian Entomologist*, *140*(6), 649–673.

Knight, H. H. (1918). Synoptic key to the subfamilies of Miridæ (Hemiptera-Heteroptera). *Journal of the New York Entomological Society*, *26*(1), 40–44.

Knutson, L., & Orth, R. E. (2001). *Sepedon mcphersoni*, n. sp., key to North American *Sepedon*, groups in *Sepedon* s.s., and intra- and intergeneric comparison (Diptera: Sciomyzidae). *Proceedings of the Entomological Society of Washington*, *103*, 620–635.

Kopp, D. D., & Yonke, T. R. (1979). A taxonomic review of the tribe Ceresini (Homptera: Membracidae). Entomological Society of America. 97 p.

Krishnankutty, S. M., Rakitov, R., & Dietrich, C. H. (2015). Taxonomy and phylogeny of the North American leafhopper genus *Cuerna* (Hemiptera: Cicadellidae). *Annals of the Entomological Society of America*, *108*(3), 339–371.

Landry, B., & Landry, J.-F. (2004). The genus *Alucita* in North America, with description of two new species (Lepidoptera: Alucitidae). *The Canadian Entomologist*, *136*(4), 553–579.

LaSalle, J. (1994). North American genera of Tetrastichinae (Hymenoptera: Eulophidae). *Journal of Natural History*, *28*(1), 109–236.

Levi, H. W. (1980). The orb-weaver genus *Mecynogea*, the subfamily Metinae and the genera *Pachygnatha*, *Glenognatha* and *Azilia* of the subfamily Tetragnathinae north of Mexico (Araneae: Araneidae). *Bulletin of The Museum of Comparative Zoology*, *149*, 1–75.

LinEpig—A resource for ID of female erigonines. (n.d.). Retrieved April 15, 2025, from https://linepig.fieldmuseum.org/

Manley, D. G., Manley, D. G., & Pitts, J. P. (2002). A key to genera and subgenera of Mutillidae (Hymenoptera) in America North of Mexico with description of a new genus. *Journal of Hymenoptera Research*, *11*(1), 72–100.

Marshall, S. A. (1982). A revision of Halidayina Duda (Diptera: Sphaeroceridae). *The Canadian Entomologist*, *114*(9), 841–847.

Marshall, S. A. (1986). A revision of the Nearctic species of the genus *Pullimosina* (Diptera, Sphaeroceridae). *Canadian Journal of Zoology*, *64*(2), 522–536.

Martin, C. H. (1957). A revision of the Leptogastrinae in the United States (Diptera, Asilidae). *Bulletin of the American Museum of Natural History*, *111*(5), 343–386.

Masner, L. (1976). Revisionary notes and keys to the world genera of Scelionidae (Hymenoptera: Proctotrupoidea). *The Memoirs of the Entomological Society of Canada*, *108*(S97), 1–87.

Masner, L., & R, J. L. G. (2002). The genera of the Diapriinae (Hymenoptera: Diapriidae) in the New World. *Bulletin of the American Museum of Natural History*, *2002*(268), 1–138.

McAlpine, J. F. (Ed.). (1987). Manual of Nearctic Diptera Vol. 1 & 2. Agriculture Canada. 674 & 668 p. https://publications.gc.ca/site/eng/9.817747/publication.html

McAtee, W. L. (1914). Key to the nearctic genera and species of Geocorinae (Heteroptera; Lygaeidae). *Proceedings of The Biological Society of Washington*, *27*, 125–136.

Melander, A. L. (1920). Synopsis of the dipterous family Psilidæ. *Psyche: A Journal of Entomology*, *27*(5), 042460.

Menard, K. L. (2015). A review of the genus *Spanagonicus* Berg (Hemiptera: Miridae: Phylinae: Nasocorini) with the description of novel antennal characters, the description of a new species from Central America, and a key to currently known taxa. *Zootaxa*, *3973*(1), 1.

Menke, A. S. (2021). The Ammophila of North & Central America (Hymenoptera, Sphecidae). Allen Press. 162 p.

Miller, M. E., Marshall, S. A., & Grimaldi, D. A. (2017). A review of the species of *Drosophila* (Diptera: Drosophilidae) and genera of Drosophilidae of northeastern North America. *Canadian Journal of Arthropod Identification*, *31*, 1–281.

Milli-PEET: Key to Millipede Orders—Field Museum. (n.d.). Retrieved April 15, 2025, from https://www.fieldmuseum.org/science/special-projects/milli-peet-class-diplopoda/milli-peet-millipedes-made-easy/milli-peet-key

Miranda, G. F. G., Young, A. D., Locke, M. M., Marshall, S. A., Skevington, J. H., & Thompson, F. C. (2013). Key to the genera of Nearctic Syrphidae. *Canadian Journal of Arthropod Identification*, *23*, 1–339.

Mlynarek, J. J., & Wheeler, T. A. (2018). Phylogeny and revised classification of the tribe Elachipterini (Diptera: Chloropidae). *Zootaxa*, *4471*(1), 1–36.

Mohamed, S. K., Gad Allah, S. M., Fadel, H. H., Radawy, R. M., & Sawaby, Rabab F. (2015). Taxonomic review of family Rhyparochromidae (Hemiptera: Lygaeoidea) from Egypt. *The Egyptian Journal of Experimental Biology*, *9*(1), 33–33.

Mohrig, W., Heller, K., Hippa, H., Vilkamaa, P., & Menzel, F. (2013). Revision of black fungus gnats (Diptera: Sciaridae) of North America. *Studia Dipterologica*, *19*, 141–286.

Nemes, S. N., & Price, D. L. (2015). Illustrated keys to the Scarabaeinae (Coleoptera: Scarabaeidae) of Maryland. *Northeastern Naturalist*, *22*(2), 318–344.

Newton, A. F., Jr. (1990). 38. Insecta: Coleoptera Staphylinidae adults and larvae. In D. L. Dindal (Ed.), *Soil Biology Guide* (pp. 1137–1174). John Wiley & Sons, Inc.

Oman, P. W. (1939). Revision of the genus *Ceratagallia* Kirkaldy (Homoptera: Cicadellidae). *Journal of the Washington Academy of Sciences*, *29*(12), 529–543.

Orozco, J. (2012). Monographic revision of the American genus *Euphoria* Burmeister, 1842 (Coleoptera: Scarabaeidae: Cetoniinae). *The Coleopterists Bulletin*, *66*(11), 1–182.

Pacheco, J. A., & Mackay, W. P. (2013). The Systematics and Biology of the New World Thief Ants of the Genus Solenopsis (Hymenoptera: Formicidae). The Edwin Mellen Press. 501

Packauskas, R. (2012). The Pentatomidae, or stink bugs, of Kansas with a key to species (Hemiptera: Heteroptera). *Great Lakes Entomologist*, *45*(3–4), 210–219.

Paiero, S. M., & Marshall, S. A. (2020). A revision of the genus *Rudolfina* Roháček (Sphaeroceridae: Limosininae). *European Journal of Taxonomy*, *593*, Article 593.

Parsons, C. T. (1975). Revision of Nearctic Mycetophagidae (Coleoptera). *The Coleopterists Bulletin*, *29*(2), 93–108.

Pereira, L., & Hoffman, R. (1993). The American species of *Escaryus*, a genus of Holarctic centipedes (Geophilomorpha: Schendylidae). *Jeffersoniana*, *3*, 1–72.

Pérusse, J. R., & Wheeler, T. A. (2000). Revision of the Nearctic species of *Lauxania* (Diptera: Lauxaniidae). *The Canadian Entomologist*, *132*(4), 411–427.

Pinto, J. D. (2006). A review of the new world genera of Trichogrammatidae (Hymenoptera). *Journal of Hymenoptera Research*, *15*, 38–163.

Platnick, N. I., & Dondale, C. D. (1992). The ground spiders of Canada and Alaska: Araneae: Gnaphosidae. Agriculture Canada. 297 p.

Platnick, N. I., & Shadab, M. U. (1981). A revision of the spider genus *Sergiolus* (Araneae, Gnaphosidae). *American Museum Novitates*, *2717*, 1–41.

Prena, J. (2009). A review of the species of *Geraeus* Pascoe and *Linogeraeus* Casey found in the continental United States (Coleoptera: Curculionidae: Baridinae). *The Coleopterists Bulletin*, *63*(2), 123–172.

Purrington, F. F., & Drake, Cathy J. (2009). A key to adult nearctic *Pasimachus* (Pasimachus) Bonelli (Coleoptera: Carabidae: Scaritini), with comments on their functional mouthpart morphology. *Entomological News*, *116*(4), 253–262.

Richman, D. B. (1989). A revision of the genus *Hentzia* (Araneae, Salticidae). *The Journal of Arachnology*, *17*(3), 285–344.

Richman, D. B., & Vetter, R. S. (2004). A review of the spider genus *Thiodina* (Araneae, Salticidae) in the United States. *The Journal of Arachnology*, *32*(3), 418–431.

Rider, D. (2012). The Heteroptera (Hemiptera) of North Dakota I: Pentatomomorpha: Pentatomoidea. *The Great Lakes Entomologist*, *45*(2), 13.

Rider, D. A. (1989). Review of the new world species of the genus *Neottiglossa* Kirby (Heteroptera: Pentatomidae). *Journal of the New York Entomological Society*, *97*(4), 394–408.

Rider, D. A., & Chapin, J. B. (1992). Revision of the genus *Thyanta* Stål, 1862 (Heteroptera: Pentatomidae) II. North America, Central America, and the West Indies. *Journal of the New York Entomological Society*, *100*(1), 42–98.

Riley, E. G. (1986). Review of the tortoise beetle genera of the tribe Cassidini occurring in America North of Mexico (Coleoptera: Chrysomelidae: Cassidinae). *Journal of the New York Entomological Society*, *94*(1), 98–114.

Roberts, A. W. R. (1930). A key to the principal families of Coleoptera in the larval stage. *Bulletin of Entomological Research*, *21*(1), 57–72.

Roháček, J., & Barber, K. N. (2016). Nearctic Anthomyzidae: A monograph of *Anthomyza* and allied genera (Diptera). *Acta Entomologica Musei Nationalis Pragae*, *56*(3), 3.

Rolston, L. H. (1978). A revision of the genus *Mormidea* (Hemiptera: Pentatomidae). *Journal of the New York Entomological Society*, *86*(3), 161–219.

Sabrosky, C. W. (1940). Twelve new North American species of *Oscinella* (Diptera, Chloropidae). *The Canadian Entomologist*, *72*(11), 214–230.

Sabrosky, C. W. (1941a). The genus *Ectecephala* in North America (Diptera, Chloropidae). *Proceedings of The Entomological Society of Washington*, *43*(4), 75–80.

Sabrosky, C. W. (1941b). The *Hippelates* flies or eye gnats: Preliminary notes. *The Canadian Entomologist*, *73*(2), 23–27.

Sabrosky, C. W. (1948). A synopsis of the Nearctic species of *Elachiptera* and related genera (Diptera, Chloropidae). *Journal of the Washington Academy of Sciences*, *38*(11), 365–382.

Scudder, G. G. E. (1999). The genus *Peritrechus* Fieber (Hemiptera: Rhyparochromidae) in North America. *Journal of the New York Entomological Society*, *107*(2/3), 268–276.

Scudder, G. G. E. (2008). Three new species of Heteroptera (Hemiptera: Rhopalidae, Rhyparochromidae) from western North America. *Proceedings of the Entomological Society of Washington*, *110*(4), 1202–1211.

Seeley, R. M. (1928). Revision of the spider genus *Tetragnatha*. *New York State Museum Bulletin*, *278*, 99–150.

Sharkey, M. (2006). Two new genera of Agathidinae (Hymenoptera: Braconidae) with a key to the genera of the New World. *Zootaxa*, *1185*(1), Article 1.

Sharkey, M., Athey, K. J., Fernandez-Triana, J. L., Penteado-Dias, A. M., Monckton, S. K., & Quicke, D. L. J. (2023). Key to the new world subfamilies of the family Braconidae (Hymenoptera). *Canadian Journal of Arthropod Identification*, *49*, 1–44.

Shultz, J. W. (2018a). A guide to the identification of the harvestmen (Arachnida: Opiliones) of Maryland. *Northeastern Naturalist*, *25*(1), 21–49.

Shultz, J. W. (2018b). A guide to the identification of the terrestrial Isopoda of Maryland, U.S.A. (Crustacea). *ZooKeys*, *801*, 207–228.

Sinada, N. A., & Blocker, H. D. (1994). Revision of the new world genus *Polyamia* (Homoptera: Cicadellidae). *Annals of the Entomological Society of America*, *87*(6), 771–794.

Slater, A., & Slater, A. (1993). A genus level revision of western hemisphere Lygaeinae (Heteroptera: Lygaeidae) with keys to species. *The University of Kansas Science Bulletin*, *55*(1), 1--56.

Slater, J. A. (1979). The systematics, phylogeny, and zoogeography of the Blissinae of the world (Hemiptera, Lygaeidae). *Bulletin of the American Museum of Natural History*, *165*, 1.

Slater, J. A., & Baranowsky, R. M. (1990). Lygaeidae of Florida (Hemiptera Heteroptera). *Arthropods of Florida and Neighboring Land Areas*, *14*, 1–232.

Slipinski, A., Tomaszewska, W., & Lawrence, J. (2009). Phylogeny and classification of Corylophidae (Coleoptera: Cucujoidea) with descriptions of new genera and larvae. *Systematic Entomology*, *34*, 409–433.

Smetana, A. (1978). Revision of the subfamily Sphaeridiinae of America north of Mexico (Coleoptera: Hydrophilidae). *The Memoirs of the Entomological Society of Canada*, *110*(S105), 1–292.

Smith, D. R. (1969). Key to genera of Nearctic Argidae (Hymenoptera) with revisions of the genera *Atomacera* Say and *Sterictiphora* Billberg. *Transactions of the American Entomological Society (1890-)*, *95*(4), 439–457.

Smith, E. H. (1985). Revision of the genus *Phyllotreta* Chevrolat of America north of Mexico. Part I. The maculate species (Coleoptera: Chrysomelidae, Alticinae). *Fieldiana*, *28*, 1–168.

Smith, I. P., & Marshall, S. A. (2004). A review of the new world genus *Pterogramma* Spuler and a revision of the *Pterogramma sublugubrinum* group (Diptera: Sphaeroceridae: Limosininae). *Contributions in Science*, *499*, 1--163.

Smith, N. J. (2009). A review of the Nearctic species of *Parammoplanus* (Pate) with descriptions of new species (Hymenoptera: Crabronidae). *The Pan-Pacific Entomologist*, *85*(3), 107–149.

Smith, T. R., Froeba, J. G., & Capinera, J. L. (2004). Key to the grasshoppers (Orthoptera: Acrididae) of Florida. *Florida Entomologist*, 537–550.

Smithers, C. N. (1990). Keys to the families and genera of Psocoptera (Arthropoda: Insecta). *Technical Reports of the Australian Museum*, *2*, 1–82.

Summers, G. (1979). An illustrated key to the chilopods of the north-central region of the United States. *Journal of the Kansas Entomological Society*, *52*(4), 690–700.

Swanson, D. (2018). A synopsis of the damsel bugs (Heteroptera: Nabidae) of Michigan. *The Great Lakes Entomologist*, *45*(1), 40-55.

Tabacaru, I., Giurginca, A., & Baba, Ș. (2018). Identification key to the Symphyla of Romania. *Travaux de l’Institut de Spéologie “Émile Racovitza,”* *LVI*, 3–23.

Talamas, E. J., Johnson, N. F., & Buffington, M. (2015). Key to Nearctic species of *Trissolcus* Ashmead (Hymenoptera, Scelionidae), natural enemies of native and invasive stink bugs (Hemiptera, Pentatomidae). *Journal of Hymenoptera Research*, *43*, 45–110.

Tatarnic, N. J., & Cassis, G. (2012). The Halticini of the world (Insecta: Heteroptera: Miridae: Orthotylinae): generic reclassification, phylogeny, and host plant associations. *Zoological Journal of the Linnean Society*, *164*(3), 558–658.

Thompson, R. G. (1979). Larvae of North American Carabidae with a key to the tribes. In T. L. Erwin, G. E. Ball, D. R. Whitehead, & A. L. Halpern (Eds.), *Carabid Beetles: Their Evolution, Natural History, and Classification* (209–291 p.). Springer Netherlands.

Thyssen, P. J. (2010). Keys for identification of immature insects. In J. Amendt, M. L. Goff, C. P. Campobasso, & M. Grassberger (Eds.), *Current Concepts in Forensic Entomology* (25–42 p.). Springer Netherlands.

Townes, H. (1981). A revision of the Serphidae (Hymenoptera). *Memoirs of the American Entomological Institute*, *32*, 1–541.

van Berge Henegouwen, A. (1989). *Sphaeridium marginatum* reinstated as a species distinct from *S. bipustulatum* (Coleoptera, Hydrophilidae). *Entomologische Berichten*, *49*(11), 168–170.

van Berge Henegouwen, A., & Foster, G. N. (2019). A new illustrated key to the British species of *Sphaeridium*, with the possibility of *S. substriatum* Faldermann, 1839 as a British species (Hydrophilidae: Sphaeridiinae). *The Coleopterist*, *28*(1), 1–12.

Van Duzee, M. C. (Millard C.), & Curran, C. H. (1934a). Key to the females of Nearctic *Dolichopus* Latreille (Diptera). *American Museum Novitates*, *684*, 1–17.

Van Duzee, M. C. (Millard C.), & Curran, C. H. (1934b). Key to the males of Nearctic *Dolichopus* Latreille (Diptera). *American Museum Novitates*, *683*, 1–26.

van Vondel, Bernhard J. (2021). Revision of the Nearctic Haliplidae (Coleoptera). *Tijdschrift Voor Entomologie*, *163*(2–3), 101–298.

Vaurie, P. (1951). Revision of the genus *Calendra* (formerly Sphenophorus) in the United States and Mexico (Coleoptera, Curculionidae). *Bulletin of the Museum of Natural History*, *98*, article 2.

Vockeroth, J. R. (2002). The flower flies of the subfamily Syrphinae of Canada, Alaska and Greenland: Diptera: Syrphidae*.* Agriculture Canada. 456 p.

Vogel, B. R. (2004). A review of the spider genera *Pardosa* and *Acantholycosa* (Araneae, Lycosidae) of the 48 contiguous United States. *The Journal of Arachnology*, *32*(1), 55–108.

Wallace, H. K., Wallace, H. K., & Exline, H. (1977). Spiders of the genus *Pirata* in North America, Central America and the West Indies (Araneae: Lycosidae). *The Journal of Arachnology*, *5*(1), 1--112.

Walley, G. S. (1929). Notes on *Homaemus* with a key to the species (Hemip., Scutelleridae). *The Canadian Entomologist*, *61*(11), 253–256.

Weirauch, C., Berenger, J.-M., Berniker, L., Forero, D., Forthman, M., Frankenberg, A., Freedman, A., Gordon, E., Hoey-Chamberlain, R., Hwang, W. S., Marshall, S. A., Michael, A., Paiero, S. M., Udah, O., Watson, C., Yeo, M., Zhang, G., & Zhang, J. (2014). An illustrated identification key to assassin bug subfamilies and tribes (Hemiptera: Reduviidae). *Canadian Journal of Arthropod Identification*, *26*, 1–115.

Werner, F. G. (1962). A revision of the Nearctic species of *Sapintus* (Coleoptera: Anthicidae). *Annals of the Entomological Society of America*, *55*(5), 492–498.

Wharton, R. A., Marsh, P. M., & Sharkey, M. J. (Eds.). (1997). Manual of the new world genera of the family Braconidae (Hymenoptera). International Society of Hymenopterists 439 p.

Wharton, R. A., & Moon, R. D. (1979). Puparia of Cyclorrhaphous Diptera from bovine dung in open pasture and rangeland in the transition zone of western North America. *Annals of the Entomological Society of America*, *72*(1), 80–89.

Wheeler, Marshall R. (1952). XI. The Drosophilidae of the Nearctic region, exclusive of the genus *Drosophila*. In Patterson, J. T., *Studies in the genetics of Drosophila. VII, Further articles on genetics, cytology and taxonomy* (pp. 163–218). University of Texas.

White, R. E. (1969). A review of the genus *Cryptocephalus* in America north of Mexico (Chrysomelidae: Coleoptera). Smithsonian Institution Press. 136 p.

Whitman-Zai, J., Francis, M., Geick, M., & Cushing, P. E. (2015). Revision and morphological phylogenetic analysis of the funnel web spider genus *Agelenopsis* (Araneae: Agelenidae). *The Journal of Arachnology*, *43*(1), 1–25.

Whitworth, T. (2006). Keys to the genera and species of blow flies (Diptera: Calliphoridae) of America north of Mexico. *Proceedings of the Entomological Society of Washington*, *108*(3), 689–725.

Wygodzinsky, P. W., & Schmidt, K. (1991). Revision of the new world Enicocephalomorpha (Heteroptera). *Bulletin of the American Museum of Natural History*, *200*, 1–265.

Yau, T., & Marshall, S. A. (2018). A revision of the genus *Bromeloecia* Spuler (Diptera: Sphaeroceridae: Limosininae). *Zootaxa*, *4445*(1), 1.
